# A Randomized Trial of Dolutegravir Plus Darunavir/Cobicistat as a Switch Strategy in HIV-1-Infected Patients With Resistance to at Least 2 Antiretroviral Classes

**DOI:** 10.1093/ofid/ofad542

**Published:** 2023-10-31

**Authors:** José R Santos, Pere Domingo, Joaquín Portilla, Félix Gutiérrez, Arkaitz Imaz, Helem Vilchez, Adrià Curran, Nieves Valcarce-Pardeiro, Antoni Payeras, Enrique Bernal, Marta Montero-Alonso, Miguel Yzusqui, Bonaventura Clotet, Sebastià Videla, José Moltó, Roger Paredes

**Affiliations:** Infectious Diseases Department and Fundació Lluita contra les Infeccions, Hospital Universitari Germans Trias i Pujol, Badalona, Catalonia, Spain; HIV Unit, Service of Internal Medicine, Hospital de la Santa Creu i Sant Pau, Barcelona, Spain; Hospital General Universitario Dr Balmis, Alicante, Spain; Infectious Diseases Unit, Department of Internal Medicine, Hospital General Universitario de Elche, Elche, Spain; University Miguel Hernández, Alicante, Spain; CIBERINFEC, ISCIII, Madrid, Spain; HIV Unit, Infectious Diseases Service, Hospital Universitari de Bellvitge, Barcelona, Spain; Infectious Diseases Unit, Internal Medicine Department, Hospital Universitari Son Espases, Fundació Institut d’Investigació Sanitària Illes Balears, Palma de Mallorca, Spain; HIV Unit, Service of Infectious Diseases, Hospital Universitari Vall d’Hebron, Barcelona, Spain; Hospital Pharmacy, Complexo Hospitalario Universitario de Ferrol, Ferrol, Spain; Service of Internal Medicine, Hospital Son Llàtzer, Palma de Mallorca, Spain; Infectious Diseases Unit, Hospital General Universitario Reina Sofía de Murcia, Murcia, Spain; Instituto Murciano de Investigación Biosanitaria, Murcia, Spain; Infectious Diseases Unit, Hospital Universitario y Politécnico La Fe, Valencia, Spain; Department of Internal Medicine, Hospital General Nuestra Señora del Prado, Talavera de la Reina, Spain; Infectious Diseases Department and Fundació Lluita contra les Infeccions, Hospital Universitari Germans Trias i Pujol, Badalona, Catalonia, Spain; IrsiCaixa AIDS Research Institute, Badalona, Spain; Pharmacology Unit, Department of Pathology and Experimental Therapeutics, School of Medicine and Health Sciences, IDIBELL, University of Barcelona, L’Hospitalet de Llobregat, Barcelona, Spain; Lluita contra les Infeccions Foundation, Badalona, Spain; Infectious Diseases Department and Fundació Lluita contra les Infeccions, Hospital Universitari Germans Trias i Pujol, Badalona, Catalonia, Spain; CIBERINFEC, ISCIII, Madrid, Spain; Infectious Diseases Department and Fundació Lluita contra les Infeccions, Hospital Universitari Germans Trias i Pujol, Badalona, Catalonia, Spain; IrsiCaixa AIDS Research Institute, Badalona, Spain; Center for Global Health and Diseases, Department of Pathology, School of Medicine, Case Western Reserve University, Cleveland, Ohio, USA

**Keywords:** darunavir/cobicistat, dolutegravir, multidrug resistance HIV-1, switch strategy

## Abstract

**Background:**

Suppressed patients with drug-resistant HIV-1 require effective and simple antiretroviral therapy to maintain treatment adherence and viral suppression.

**Methods:**

This randomized, open-label, noninferiority, multicenter pilot study involved HIV-infected adults who met the following criteria: confirmed HIV-1 RNA <50 copies/mL for ≥6 months preceding the study randomization, treatment with at least 3 antiretroviral drugs, and a history of drug resistance mutations against at least 2 antiretroviral classes but remaining fully susceptible to darunavir (DRV) and integrase inhibitors. Participants were randomized 1:1 to switch to dolutegravir (DTG; 50 mg once per day) plus DRV boosted with cobicistat (DRV/c; 800/150 mg once per day; 2D group) or continue with their baseline regimen (standard-of-care [SOC] group). The primary endpoint was the proportion of patients with HIV-1 RNA <50 copies/mL at week 48 relative to time to loss of virologic response, with a noninferiority margin set at −12.5%. Virologic failure was defined as confirmed HIV-1 RNA ≥50 copies/mL or a single determination of HIV-1 RNA >50 copies/mL followed by antiretroviral therapy discontinuation.

**Results:**

Forty-five participants were assigned to the 2D group and 44 to the SOC group. Time to loss of virologic response showed no difference in the proportion maintaining HIV-1 RNA <50 copies/mL at week 48: 39 of 45 (86.7%; 95% CI, 73.21%–94.95%) in the 2D group vs 42 of 44 (95.4%; 95% CI, 84.53%–99.44%) in the SOC group (log-rank *P* = .159) with an estimated difference of −8.7 (95% CI, −22.72 to 5.14). Only 2 (4.5%) in the SOC group experienced virologic failure, and 3 participants from the 2D group experienced adverse events leading to treatment discontinuation.

**Conclusions:**

In suppressed patients with at least 2 resistant antiretroviral classes, noninferiority could not be demonstrated by fully active DRV/c plus DTG. Nevertheless, there were no unexpected adverse events or virologic failure. DRV/c plus DTG may be considered a once-daily therapy option only for well-selected patients.

**Clinical Trials Registration.** ClinicalTrials.gov (NCT03683524).

Simple, effective, and well-tolerated antiretroviral therapy (ART) combinations are needed to maintain lifelong HIV-1 suppression in patients with high therapeutic experience and multidrug resistant HIV-1. These individuals often achieve virus suppression through complex ART combinations, which are difficult to maintain in the long run due to pill fatigue and poor treatment adherence. Dual ART based on boosted protease inhibitors plus integrase strand transfer inhibitors (INSTIs) has been evaluated in clinical trials as a second-line salvage strategy in those with HIV harboring drug resistance mutations (DRMs) [1] but not as a maintenance strategy in patients with archived DRM.

Darunavir (DRV) boosted with ritonavir (DRV/r) or cobicistat (DRV/c) and dolutegravir (DTG) can be given once daily, are well tolerated, and have a high genetic barrier to resistance. DRV and DTG have demonstrated antiviral efficacy in patients with limited therapeutic options [2–8].

DTG's major and minor metabolic pathways are UDP-glucuronosyltransferase 1A1 and cytochrome 3A4, respectively. Since ritonavir is a UDP-glucuronosyltransferase inducer, coadministration of DTG with ritonavir can decrease trough concentrations of DTG by nearly 40%, which may lead to inappropriately low concentrations of DTG in some patients. By contrast, cobicistat does not induce glucuronidation [9], and DTG trough concentration levels are not affected by its coadministration with DRV/c [10].

Here, we evaluated the efficacy and safety of DTG plus DRV/c as a once-daily maintenance strategy vs continuing the previous ART in patients who were highly treatment experienced with HIV-1 resistant to drugs but not INSTIs or DRV.

## METHODS

### Study Design

This was an open-label, randomized, noninferiority, and multicenter pilot clinical trial to evaluate the efficacy of DTG plus DRV/c as a once-daily simplification strategy as compared with the continuation of the current ART in maintaining virologic suppression (RNA HIV-1 <50 copies/mL) in participants who were highly ART experienced and harboring archived DRM against at least 2 antiretroviral classes.

Study participants were randomized 1:1 to receive dual therapy based on DTG (50 mg) plus DRV/c (800/150 mg) once daily (the 2D arm) or a continuation of their current stable and standard-of-care (SOC) ART (the control arm). The randomization list was created by using a uniform distribution and assigning a range of values to each group. The assignment was made by REDCap's randomization module. The study duration was 52 weeks, which included a 4-week screening period plus a 48-week follow-up period.

### Patient Consent and Ethical Statements

The study was approved by the Ethics Committee of the Hospital Universitari Germans Trias i Pujol (AC-18-074-HGT-CEIM) and by the Spanish Regulatory Authorities, and it was conducted according to the stipulations of the Declaration of Helsinki (Brazil, 2013) and local personal data protection law (LOPD 15/1999). All patients provided their written informed consent for participation. The study is registered at ClinicalTrials.gov (NCT03683524).

### Study Population

Eligible participants were HIV-infected adults treated with at least 3 antiretroviral drugs (protease inhibitors, nonnucleoside reverse transcriptase inhibitors, nucleoside reverse transcriptase inhibitors, integrase inhibitors, or CCR5 receptor antagonists) and with confirmation of suppressed HIV-1 RNA <50 copies/mL during at least 6 months preceding randomization. Patients had to have historical genotypic evidence of DRM against ≥2 antiretroviral classes according to the Stanford DB (version 9.0; https://hivdb.stanford.edu). However, their virus had to remain fully susceptible to DRV (Stanford DB score <15) and lack all the following INSTI-associated DRMs: T66A/I/K, L74M, E92G/Q, G118R, T97A, F121Y, E138A/K/T, G140A/C/S, Y143A/C/G/R/H/S, S147G, Q148H/K/R, N155H, S230R, and R263K. Participants were considered not eligible if they had a history of poor adherence or virologic failure while receiving integrase inhibitors; had concomitant treatments with potential drug interactions with DRV/c or DTG; were hepatitis B surface antigen positive; or had unstable liver disease, severe hepatic impairment (Child-Pugh class C), or hepatitis C coinfection that would require therapy during the study.

### Outcomes

The primary study outcome was the proportion of participants with HIV-1 RNA <50 copies/mL at week 48 with an analysis based on time to loss of virologic response (TLOVR). Secondary endpoints included the proportion of participants with HIV-1 RNA <50 copies/mL at week 48 per the Food and Drug Administration (FDA) snapshot algorithm; the percentage developing ART-associated adverse events leading to treatment discontinuation; changes in CD4^+^ cell counts and biochemical parameters during the follow-up; and the emergence of new DRMs in the protease and integrase genes of the HIV-1 in participants experiencing virologic failure.

ART adherence was calculated as follows: (number of capsules taken / number of capsules prescribed) × 100. Concentrations of DTG and DRV/c in plasma were determined at week 4 in all patients from the 2D arm and in participants experiencing virologic failure. To this end, blood samples for DTG, DRV, and cobicistat were collected in 5-mL tubes containing potassium and EDTA. Plasma was isolated by centrifugation (1900*g* for 15 minutes) and stored at −20 °C until analysis. DTG and DRV concentrations in plasma were determined with ultrahigh-performance liquid chromatography with tandem mass spectrometry according to a validated method [11].

### Statistical Analysis

The sample size was estimated to provide 80% power with a 1-sided alpha value of 0.05 to detect 95% efficacy (according to the TLOVR analysis) in the group treated with DTG plus DRV/c and in the SOC arm with a noninferiority limit of −12.5%. A final sample size of 102 participants (51 per arm) was planned to account for a 5% discontinuation rate during the study follow-up. However, patient inclusion had to be stopped after 109 weeks because there were no more eligible patients in the participating centers. There were no more eligible patients mainly because of participation in other clinical and observational studies, treatment with dual therapies different from the one being studied, the presence of mutations in the protease conferring resistance to DRV (≥15 points according to the Stanford DB), genotypic confirmation or suspicion of resistance to integrase inhibitors, and finally the onset of the SARS-CoV-2 pandemic.

Virologic failure was defined as detection of HIV-1 RNA ≥50 copies/mL at 2 consecutive visits measured within 2 to 4 weeks or as a single determination of HIV-1 RNA ≥50 copies/mL followed by premature treatment discontinuation. A genotyping test was performed at virologic failure confirmation. If adverse events resulted in treatment discontinuation or dropout, they were treated as virologic failure for efficacy assessment. The Kaplan-Meier estimation was used to describe TLOVR analysis for all participants and by treatment arm.

Data from archived genotyping tests, viral tropism, and previous exposure to antiretroviral drugs were collected from electronic history records in the different participating centers. Demographics and clinical parameters were summarized with mean (SD), median (IQR), or frequency (percentage), as appropriate. Frequency distributions for categorical variables were compared with chi-square or Fisher exact tests. Parametric tests (*t* test, nonpaired, or paired) or nonparametric tests (Mann-Whitney, Kruskal-Wallis, Wilcoxon, or Friedman) were used to compare continuous variables, depending on their distribution and the nature of the comparison (paired or independent).

For the primary efficacy analysis, the noninferiority hypothesis test was based on a 2-sided 95% CI computed with a continuity-corrected *Z* statistic for the difference (DTG plus DRV/c – SOC) of the proportion of participants with HIV-1 RNA <50 copies/mL, and its 95% CI was calculated. Noninferiority was concluded if the upper limit of the 2-sided 95% CI was <−12.5%.

The proportion with HIV-1 RNA <50 copies/mL at week 48 according to the FDA snapshot was evaluated. In the FDA snapshot analysis, participants were classified according to 3 outcomes:

 Responders: HIV-1 RNA <50 copies/mL at week 48

 Nonresponders: HIV-1 RNA ≥50 copies/mL at week 48—participants with virologic failure or blips (in window) or participants who discontinued the study drug because of adverse events, death, or other reasons before week 48 with last available HIV-1 RNA ≥50 copies/mL

 No virologic data: participants who discontinued the study drug before week 48 for reasons other than low efficacy, including adverse event and death with last available HIV-1 RNA <50 copies/mL, and participants who were still taking the study drug but for whom HIV-1 RNA data were missing at week 48

The difference in response rates and *P* value of the snapshot analysis were calculated by a dichotomized response: HIV-1 RNA <50 copies/mL at week 48 vs HIV-1 RNA ≥50 copies/mL and no virologic data at week 48.

Statistical analysis was performed with SPSS version 15.0 (IBM) and R (R Foundation for Statistical Computing). Differences were considered statistically significant at *P* < .05.

## RESULTS

Between December 2018 and January 2021, 96 participants were randomized from 12 HIV care centers across Spain. One individual in each group withdrew his or her informed consent before baseline. In addition, 3 participants in the 2D group and 2 in the SOC group were excluded before baseline due to protocol violation. These 7 patients did not receive the study treatment. Therefore, the efficacy analysis set and safety analysis set included 89 participants: 45 in the 2D group and 44 in the SOC group (Supplementary Figure 1).

Demographics and clinical characteristics were well balanced between groups at baseline (Table 1). The median (IQR) age was 55 years (50–60); 68 (76.4%) were male; and the median time since HIV diagnosis was 25 years (23.0–28.0). Participants had a median 3 (2–8) and 5 (4–7) associated DRMs in the genes of protease and reverse transcriptase, respectively (Supplementary Table 1). Before randomization, 61 (68%) and 59 (66%) participants were taking boosted DRV or INSTIs. In addition, 33 (37.1%) were following twice-daily regimens and had previously taken a median 13 (10–17) antiretroviral drugs (Supplementary Table 2).

**Table 1.** Baseline Characteristics (N = 89)

	Arm, No. (%) or Median (IQR)	
	SOC (Control; n = 44)	2D (DRV/c + DTG; n = 45)
Sex: men	32 (72.7)	36 (80.0)
Age, y	55 (50–60)	55 (50–61)
Time since HIV diagnosis, y	25 (23–28)	25 (23–29)
Time on virologic suppression, y	4 (1–9)	5 (2–9)
Hepatitis C virus coinfection	5 (11.4)	6 (13.3)
Transmission route		
MSW	13 (29.5)	15 (33.3)
MSM	16 (36.4)	18 (40.0)
IVDU	12 (27.3)	10 (22.2)
Others	3 (4.8)	2 (4.5)
Previous exposure to antiretroviral drugs at baseline		
Total	13 (10–17)	14 (10–17)
Protease inhibitors	4 (2–6)	4 (2–6)
NRTIs	6 (5–7)	7 (5–7)
NNRTIs	2 (1–2)	2 (1–2)
Integrase inhibitors	1 (1–2)	1 (1–2)
ART at study entry		
Darunavir^a^	28 (63.6)	33 (73.3)
DRV/r^b^	15 (34.1)	13 (28.9)
DRV/c	13 (29.5)	20 (44.4)
Other PI	3 (6.8)	…
3TC/FTC	27 (61.4)	26 (57.8)
TDF/TAF	23 (52.3)	17 (37.8)
ABC	7 (15.9)	9 (20.0)
ETR	9 (20.5)	13 (28.9)
RAL	12 (27.3)	17 (37.8)
ELV/c	4 (9.1)	1 (2.2)
DTG QD	13 (29.5)	17 (37.8)
Twice-daily ART regimens	17 (38.6)	16 (35.6)
DRV/r BID	9 (20.5)	6 (13.3)
ETR BID	9 (20.5)	13 (28.9)
RAL BID	12 (27.3)	16 (35.6)
No. of daily tablets	5 (5–7)	3 (5–7)
CD4^+^, cells/mm^3^		
Nadir	147 (61–217)	152 (77–269)
Count	607 (429–961)	623 (493–901)
Associated mutations at baseline		
Protease	3 (2–7)	4 (2–8)
Major protease	1 (0–2)	2 (0–3)
Reverse transcriptase	3 (1–5)	5 (4–7)
TAM	3 (0–4)	3 (0–4)
NNRTI	2 (1–2)	2 (1–3)
Prior enfuvirtide failure	5 (11.4)	7 (15.6)
Resistant ART classes ^c^		
2	35 (79.5)	33 (73.3)
3	9 (20.5)	12 (26.7)
Viral tropism		
CXCR4	8 (18.2)	3 (6.7)
CCR5	8 (18.2)	6 (13.3)
NA	28 (63.6)	36 (80.0)

Abbreviations: 3TC, lamivudine; ABC, abacavir; ART, antiretroviral therapy; BID, twice a day; DRV/c, darunavir/cobicistat; DRV/r, darunavir/ritonavir; DTG, dolutegravir; ELV/c, elvitegravir/cobicistat; ETR, etravirine; FTC, emtricitabine; IVDU, intravenous drug user; MSM, men who have sex with men; MSW, men who have sex with women; NA, not available; NNRTI, nonnucleoside reverse transcriptase inhibitor; NRTI, nucleoside reverse transcriptase inhibitor; PI, protease inhibitor; QD, once a day; RAL, raltegravir; SOC, standard of care; TAF, tenofovir alafenamide; TAM, thymidine analog mutation; TDF, tenofovir disoproxil fumarate.

^a^Including ritonavir and cobicistat.

^b^Including QD and BID doses.

^c^According to Stanford DB (version 9.0; https://hivdb.stanford.edu).

**Figure 1.**
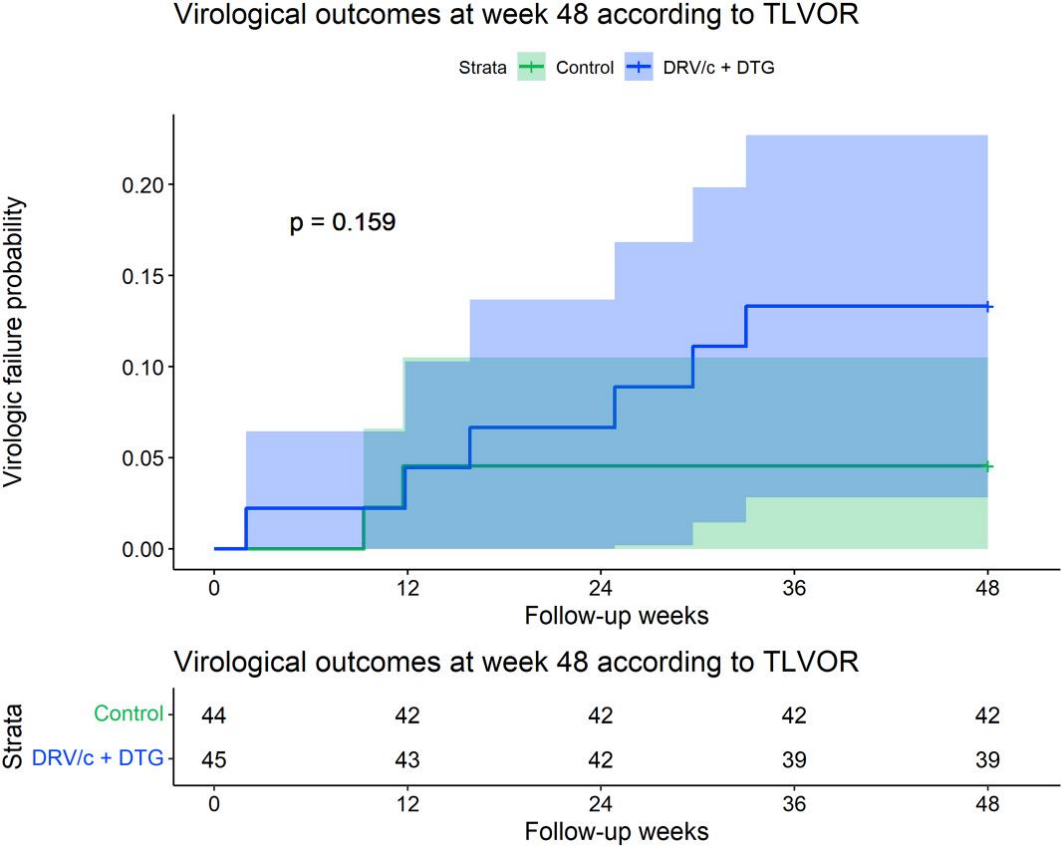
Virologic outcomes at week 48 according to TLOVR. Virologic failure was defined as detection of HIV-1 RNA ≥50 copies/mL at 2 consecutive visits measured within 2 to 4 weeks or by a single determination of HIV-1 RNA ≥50 copies/mL followed by premature treatment discontinuation. In case of an adverse event leading to treatment discontinuation or dropout, it was treated as virologic failure for efficacy. Kaplan-Meier estimation was used to describe TLOVR analysis for all participants and by treatment arms. DRV/c, darunavir with cobicistat; DTG, dolutegravir; TLOVR, time to loss of virologic response.

No virologic failures occurred in the 2D group. In contrast, 2 (4.5%) met the criteria for confirmed virologic failure at weeks 12 and 24 in the SOC group. Information about the 2 participants who experienced virologic failure is detailed in the Supplementary Table 3. In the 2D group, 3 patients experienced adverse events leading to treatment discontinuation, and an additional 3 patients were lost to follow-up, as opposed to none in the SOC group. No patients in the 2D group who had switched from a twice-daily regimen experienced discontinuations. These discontinuations due to adverse events were observed only among participants who had switched from once-daily treatment, with 2 of them receiving a coformulated single-tablet regimen. According to the TLOVR analysis (Figure 1), there were no differences in the proportion maintaining HIV-1 RNA <50 copies/mL at week 48 (95.5% vs 86.7%, log rank *P* = .159). The estimated difference in the proportion of virologic failure between the experimental group and the control was −8.7 (95% CI, −22.72 to 5.14). Noninferiority could not be demonstrated, as the upper confidence interval of the difference exceeds the noninferiority limit of −12.5%. Similarly, the FDA snapshot analysis (n = 89) showed no differences in the proportion of patients maintaining virologic suppression in both arms (Table 2; *P* = .147). Three (6.7%) and 5 (11.4%) participants in the 2D and SOC groups, respectively, experienced blips during the follow-up period (*P* = .480).

**Table 2.** Virologic Outcomes at Week 48 According to a Food and Drug Administration Snapshot Analysis

	Arm, No. (%, 95% CI)		
	SOC (Control; n = 44)	2D (DRV/c + DTG; n = 45)	*P* Value
Success, VL <50 copies/mL	42 (95.5, 89.3–100.0)	39 (86.7, 76.7–96.6)	.147
Nonresponders, VL ≥50 copies/mL	2 (4.5, 0–10.7)	…	.148
Virologic failure	2 (4.5, 0–10.7)	…	
VL ≥50 copies/mL (in window)	…	…	
No data	…	6 (13.3, 3.4–23.2)	.012
Discontinuation due to AEs	…	3 (6.7, .6–13.9)	
Missing	…	…	
Loss to follow-up	…	3 (4.5, 0–13.9)	
M = F^a^	42 (95.5, 89.3–100.0)	39 (86.7, 76.7–96.6)	.147

Abbreviations: AE, adverse event; DRV/c, darunavir/cobicistat; DTG, dolutegravir; SOC, standard of care; VL, viral load.

^a^Missing equal to failure of efficacy analysis.

There were no differences between groups in mean ART adherence at 48 weeks (100% in the 2D group vs 99.6% in the SOC group, *P* = .334). The median (IQR) change from baseline in CD4^+^ cell count at week 48 was −1.0 cells/mm^3^ (−2.9 to 0.5) in the 2D arm and 0.4 cells/mm^3^ (−2.7 to 2.8) in the SOC arm (*P* = .130). Regarding the lipid profile, the median change in total cholesterol from baseline was −0.8 mg/dL (−19.3 to 26.0) in the 2D group and 3.00 mg/dL (−10.0 to 15.5) in the SOC group (*P* = .970).

Median changes in low- and high-density lipoprotein cholesterol were 0.0 mg/dL (−26.6 to 24.0) and −1.1 mg/dL (−6.4 to 4.4) in the 2D group and −2.3 mg/dL (−19.0 to 16.0) and −2.0 mg/dL (−5.4 to 2.8) in the SOC group (*P* = .590 and *P* = .740), respectively. Triglyceride levels did not show significant changes, with a median change of −7.0 mg/dL (−35.3 to 60.0) in the 2D group and 10.0 mg/dL (−17.3 to 40.6) in the SOC group (*P* = .570). In addition, the number of patients taking lipid-lowering drugs increased from 12 to 13 in the 2D group and from 14 to 16 in the SOC group. Similarly, creatinine plasma levels and estimated glomerular filtration rate remained stable during the study period without significant differences between groups (Table 3, Supplementary Figure 2).

**Table 3.** Changes in Lipid, CD4+, Creatinine, and CKD-EPI

	Arm, Median (IQR)							
	SOC (Control; n = 44)			2D (DRV/c + DTG; n = 45)			*P* Value	
	Baseline	Week 48	*P* Value ^a^	Baseline	Week 48	*P* Value ^a^	Baseline	Week 48
Cholesterol, mg/dL								
Total	198.0 (169.0–229.0)	205.8 (173.2–234.5)	.530	194.6 (170.1–225.5)	200.0 (167.2–228.0)	.560	.510	.429
LDL	128.0 (110.6–149.5)	126.3 (100.8–148.8)	.498	119.0 (99.0–141.0)	119.0 (103.3–143.3)	.660	.323	.511
HDL	47.0 (36.8–62.9)	44.4 (34.3–55.5)	.120	47.3 (39.5–58.0)	48.3 (38.8–58.3)	.424	.826	.429
Triglycerides, mg/dL	133.0 (92.5–191.8)	152.0 (109.5–219.8)	.236	125.7 (97.5–192.0)	136.0 (93.0–186.2)	.666	.743	.233
Creatinine, mg/dL	0.86 (0.74–0.98)	0.90 (0.72–1.06)	.355	0.90 (0.78–1.06)	0.99 (0.82–1.12)	.003	.582	.176
CKD-EPI, mL/min	80.0 (67.9–87.6)	74.1 (67.5–80.0)	.414	76.3 (67.0–82.9)	76.0 (57.0–82.2)	.012	0.370	.927
CD4 + count, cells/mm^3^	607 (429–961)	586 (440–817)	.811	623 (493–901)	644 (497–911)	.446	.761	.644

Abbreviations: CKD-EPI, Chronic Kidney Disease Epidemiology Collaboration; DRV/c, darunavir/cobicistat; DTG, dolutegravir; HDL, high-density lipoprotein; LDL, low-density lipoprotein; SOC, standard of care.

^a^Intragroup comparisons.

**Figure 2.**
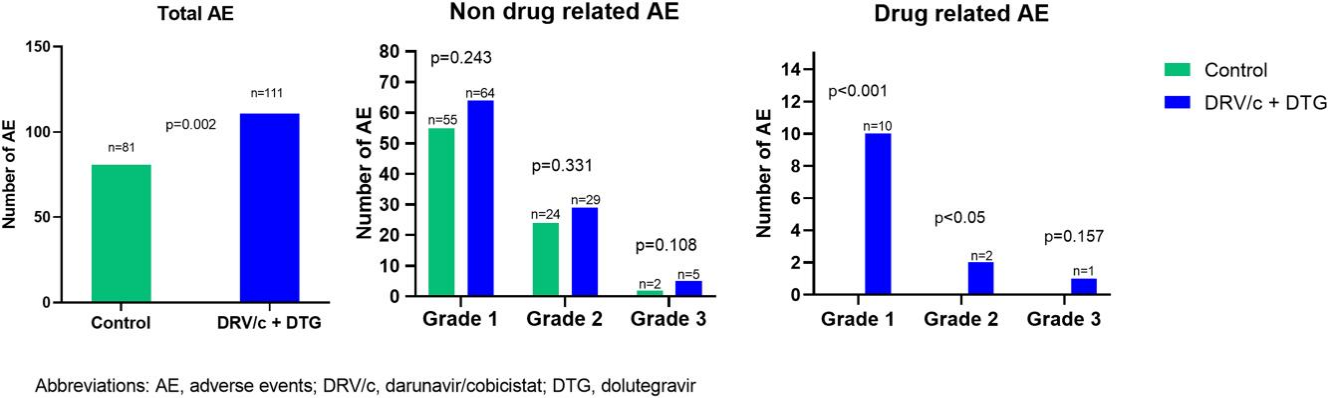
Adverse events during follow-up. Forty-one (91.1%) of 45 patients in the 2D group and 32 (72.7%) of 44 in SOC group had at least 1 adverse event. A total of 111 adverse events in the 2D group and 81 in the SOC group (*P* = .002) were documented. There were no serious adverse events. DTG + DRV/c–related adverse events were observed in 13 participants, being mild or moderate. Three of them developed adverse events leading to treatment discontinuation. No drug-related adverse events or events leading to treatment discontinuation were observed in the SOC group. 2D, DTG + DRV/C; AE, adverse event; DRV/c, darunavir with cobicistat; DTG, dolutegravir; SOC, standard of care.

Forty-one (91.1%) of 45 patients in 2D group and 32 (72.7%) of 44 in SOC group had at least 1 adverse event. A total of 111 adverse events in the 2D arm and 81 in the SOC arm (*P* = .002) were documented (Figure 2). There were no serious adverse events, AIDS-defining events, or deaths during the study. DTG plus DRV/c–related adverse events were observed in 13 participants, being mild or moderate. Three of these patients developed adverse events leading to treatment discontinuation. No drug-related adverse events or events leading to treatment discontinuation were observed in the SOC group. Two reported grade II arthralgia and rash and 1 had grade III diarrhea, all during virologic suppression. Prior ART for these 3 participants before their randomization into the 2D group was as follows: elvitegravir/cobicistat/emtricitabine/tenofovir alafenamide; DRV/c with abacavir/lamivudine; and a once-daily combination of DRV/r, DTG, and maraviroc. Ten participants experienced 9 drug-related adverse events: asthenia, drowsiness, dizziness, headache, general malaise, weight increase, night sweats, diarrhea, and salivary gland enlargement. Non–drug-related grade I, II, and III adverse events were similar in both groups (*P* > .05; Figure 2).

**Figure 3.**
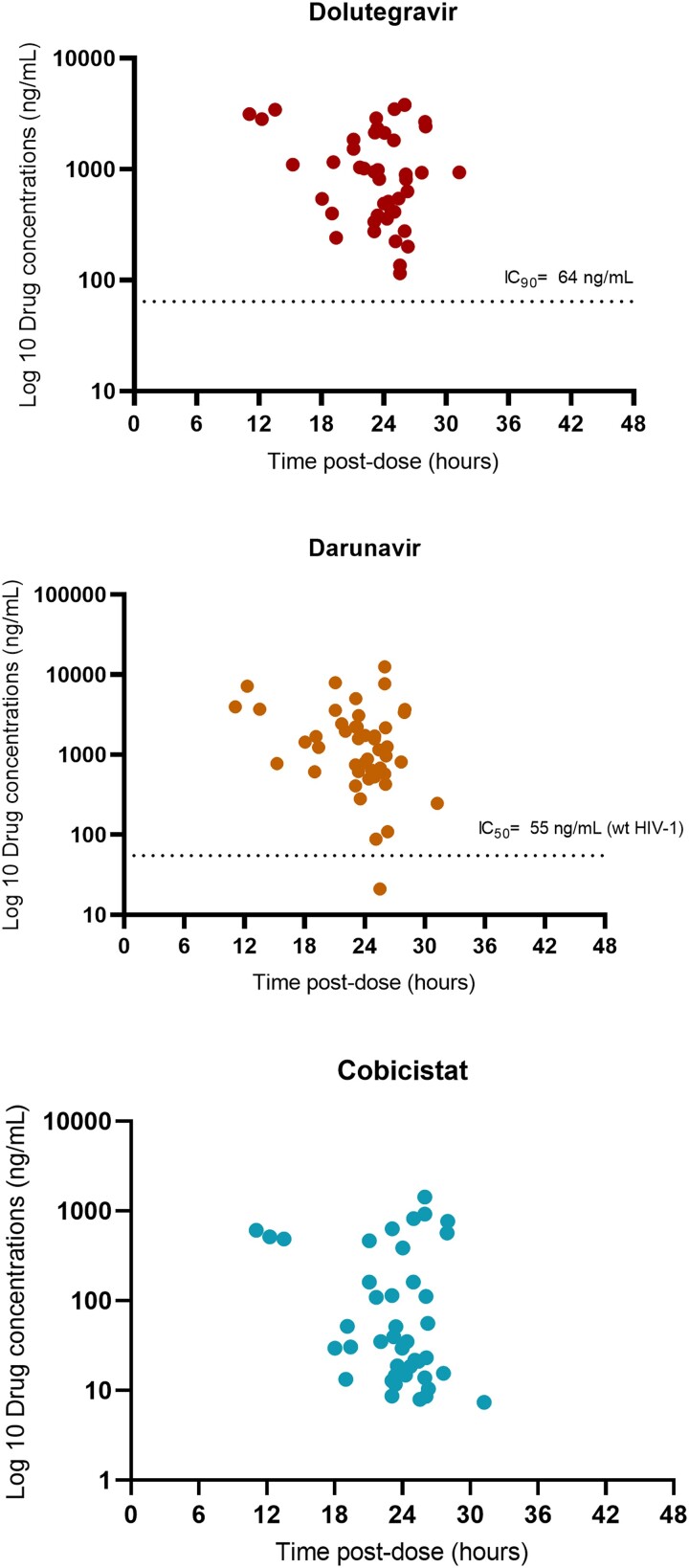
Concentrations of DTG, DRV, and cobicistat in the plasma of participants allocated to the 2D arm. Concentrations of DTG and DRV/c in plasma were determined at week 4 in all patients from the 2D arm and in those experiencing virologic failure. Blood samples for DTG, DRV, and cobicistat were collected in 5-mL tubes containing potassium and EDTA. Plasma was isolated by centrifugation (1900*g* for 15 minutes) and stored at −20 °C until analysis. DTG and DRV concentrations in plasma were determined with ultrahigh-performance liquid chromatography with tandem mass spectrometry according to a validated method [11]. 2D, DTG + DRV/C; AE, adverse event; DRV/c, darunavir with cobicistat; DTG, dolutegravir.

Trough concentrations (20–28 hours after dosing) in plasma were available for all 45 selected participants allocated to the 2D arm (Figure 3). Mean (SD) DTG, DRV, and cobicistat trough concentrations were 1317 ng/mL (1098), 2131 (2452), and 202 (317.1), respectively. DTG concentrations were above the protein-adjusted IC_90_ (90% inhibitory concentration) [12] in all cases. DRV concentrations were above the protein-adjusted IC_50_ for wild type HIV in all but 1 case [13]. No adherence issues or drug-drug interactions could be identified, and plasma viral load remained undetectable during the entire follow-up period in this participant.

## DISCUSSION

This study shows that dual therapy with DRV/c plus DTG maintains viral suppression in patients who are well suppressed, adherent to ART, and highly experienced and are harboring archived DRM against at least 2 antiretroviral classes but fully active DRV and INSTIs. This regimen may be a valid option for simplifying ART in highly experienced patients with suppressive but complex ART regimens that could cause pill fatigue and adherence concerns, as well as individuals taking drugs that may have reduced long-term effectiveness due to the presence of archived DRMs, regardless of the regimen's complexity.

In our study, there were no significant differences in virologic efficacy in the TLOVR or FDA snapshot analyses. While there were 2 virologic failures in the SOC arm, none was observed in the 2D arm. This is consistent with previous studies of protease inhibitors plus integrase inhibitors or lamivudine as first-line or simplification therapy [14, 15]. Unlike ours, previous studies excluded patients with DRM or previous virologic failure. The absence of virologic failures in the 2D group is also in line with the high genetic barrier of DTG and DRV [2, 6, 7, 16].

The efficacy of DTG and DRV has been well demonstrated in randomized clinical trials in early and advanced salvage ART, as well as a simplification in highly experienced patients harboring DRM [3–6, 17]. Until now, however, no clinical trials had evaluated this combination in patients with multidrug-resistant HIV-1. Our findings are highly consistent with previous observational [18–22], uncontrolled, single-arm, and mostly retrospective studies [18–20]. Such studies evaluated heterogeneous patient populations, including failing and well-suppressed cases [18, 19, 21], and allowed the use of other boosted protease inhibitors different from DRV [18], twice-a-day administration of DRV or DTG [20, 21], and cobicistat and ritonavir as pharmacologic boosters in the same study [18, 20–22].

Before randomization, 57% and 76% of the 2D arm and SOC arm were taking integrase inhibitors, respectively. In addition, 71% of patients in the SOC group were undergoing treatment with protease inhibitors, mainly DRV, while 73% of patients in the 2D group were taking boosted DRV before randomization. This could be the main reason why we did not observe significant differences between groups in the fasting lipid profile, plasmatic creatinine levels, and glomerular filtration rate, despite the use of cobicistat and DTG. Additionally, no additive effect was observed in these renal parameters due to the combined use of DTG and cobicistat in the same regimen, nor were any cases observed of dual-therapy discontinuation for worsening renal function or in the fasting lipid profile. These findings are concordant with clinical and observational studies in which DTG and DRV/c individually or in combination have been shown to be safe and well tolerated [6, 19, 22].

There were a high proportions of participants taking boosted DRV and INSTIs before randomization in the 2D group, including 44% with DRV/c, 29% with DRV/r, and 38% with DTG. Notably, there were more overall and drug-related adverse events in the 2D group, although they were mainly grade I and II. These safety results, however, should be interpreted with caution due to the open label design. Yet, they are not surprising, since the change to a new treatment regimen is usually associated with a higher risk of experiencing adverse events or tolerance problems. Nonetheless, it is worth noting that adverse events leading to treatment discontinuation were infrequent. Only 3 participants undergoing dual therapy with DTG plus DRV/c discontinued their ART due to arthralgia, diarrhea, and rash. In these cases, the switch to DRV/c plus DTG involved some ART changes. The patient experiencing arthralgia started both drugs for the first time. In the case of the one who discontinued due to diarrhea, ritonavir was replaced with cobicistat as the booster for DRV. For the individual who experienced rash, abacavir/lamivudine was substituted with DTG. These are well-known adverse events related to these drugs [23], and unexpected adverse events were not observed.

Our study has a number of limitations. The enrolment of patients had to be stopped due to the lack of eligible participants. Therefore, the study was underpowered to detect 90% efficacy and demonstrate the noninferiority of DTG plus DRV/c dual therapy vs maintenance of ART. In addition, the study may have introduced selection biases based on its open design and the fact that good ART adherence prior to study initiation was assumed but not measured; as such, safety and efficacy results must be interpreted with caution. Nevertheless, to our knowledge, this is the first randomized study to compare a dual therapy made up of DTG and DRV/c as a simplification strategy in patients infected with HIV who had high ART experience and resistance to at least 2 classes of antiretroviral drugs but with DTG and DRV still fully active. In addition, the study provides some insights into the main safety and pharmacokinetic issues. Similar to observations in healthy volunteers [10], our data support the claim that the combination of DTG plus DRV/c does not have a significant drug-drug interaction, since it maintains drug levels over the recommended IC_90_ for DTG [12] and IC_50_ for DRV [13] in all patients. Finally, our results might be of interest for clinicians who treat patients harboring DRM, including those who work in settings where there is a high prevalence of DRM [24].

In summary, while noninferiority could not be demonstrated and no specific advantage in terms of tolerance was observed, dual therapy with DTG plus DRV/c appeared to be a safe approach. It may maintain viral suppression without the development of virologic failures or unexpected adverse events in patients highly experienced with resistance to at least 2 antiretroviral classes, as long as they retain DRV- and INSTI-susceptible HIV-1. To minimize the risk of virologic failure, proper selection of candidates for simplification with this once-daily strategy is mandatory.

## Supplementary Data

Supplementary materials are available at *Open Forum Infectious Diseases* online. Consisting of data provided by the authors to benefit the reader, the posted materials are not copyedited and are the sole responsibility of the authors, so questions or comments should be addressed to the corresponding author.

## Supplementary Material

ofad542_Supplementary_Data

## Notes

***Acknowledgments**.* We are grateful to Michael Kennedy-Scanlon for editorial assistance; Anna García, Pau Satorra Herbera, and Nuria Pérez-Alvarez for their support in the statistical analysis; and Helena Pera and Jessica Toro for their assistance in coordinating and recording all data. Finally, we thank the patients who have participated and made this study possible.

***Author contributions***. J. R. S., J. M. and R. P. conceived and contributed to the study design. J. R. S., J. M., and R. P. drafted the manuscript. S. V. assisted in the study methodology. J. R. S., P. D., J. P., F. G., A. I., H. V., A. C., N. V.-P., A. P., E. B., M. M.-A., M. Y., B. C., J. M., and R. P. assisted in the selection of participants and were involved in critically revising the manuscript for important intellectual content.

***Disclaimer.*** The funders had no role in study design, data collection and analysis, decision to publish, or preparation of the manuscript. The authors had full access to the data, and the corresponding author had the final responsibility for submitting the manuscript for publication.

***Financial support.*** This work was supported through a researcher-initiated grant from ViiV Healthcare; by Fundació Lluita contra les Infeccions; and by the Spanish AIDS Network Red Temática Cooperativa de Investigación en SIDA (RD06/0006).

## References

[1] Boyd MA, Kumarasamy N, Moore CL, et al Ritonavir-boosted lopinavir plus nucleoside or nucleotide reverse transcriptase inhibitors versus ritonavir-boosted lopinavir plus raltegravir for treatment of HIV-1 infection in adults with virological failure of a standard first-line ART regimen (SECOND). Lancet 2013; 381:2091–9. 23769235 10.1016/S0140-6736(13)61164-2

[2] Moltó J, Valle M, Santos JR, et al Treatment simplification to once daily darunavir/ritonavir guided by the darunavir inhibitory quotient in heavily pretreated HIV-infected patients. Antivir Ther 2010; 15:219–25. 20386077 10.3851/IMP1519

[3] Madruga JV, Berger D, McMurchie M, et al Efficacy and safety of darunavir-ritonavir compared with that of lopinavir-ritonavir at 48 weeks in treatment-experienced, HIV-infected patients in TITAN: a randomised controlled phase III trial. Lancet 2007; 370:49–58. 17617272 10.1016/S0140-6736(07)61049-6

[4] Katlama C, Clotet B, Mills A, et al Efficacy and safety of etravirine at week 96 in treatment-experienced HIV type-1–infected patients in the DUET-1 and DUET-2 trials. Antivir Ther 2010; 15:1045–52. 21041921 10.3851/IMP1662

[5] Clotet B, Bellos N, Molina J-M, et al Efficacy and safety of darunavir-ritonavir at week 48 in treatment-experienced patients with HIV-1 infection in POWER 1 and 2: a pooled subgroup analysis of data from two randomised trials. Lancet 2007; 369:1169–78. 17416261 10.1016/S0140-6736(07)60497-8

[6] Castagna A, Maggiolo F, Penco G, et al Dolutegravir in antiretroviral-experienced patients with raltegravir- and/or elvitegravir-resistant HIV-1: 24-week results of the phase III VIKING-3 study. J Infect Dis 2014; 210:354–62. 24446523 10.1093/infdis/jiu051 PMC4091579

[7] Cahn P, Fourie J, Grinsztejn B, et al Week 48 analysis of once-daily vs twice-daily darunavir/ritonavir in treatment-experienced HIV-1-infected patients. AIDS 2011; 25:929–39. 21346512 10.1097/QAD.0b013e328345ee95

[8] Cahn P, Pozniak AL, Mingrone H, et al Dolutegravir versus raltegravir in antiretroviral-experienced, integrase-inhibitor-naive adults with HIV: week 48 results from the randomised, double-blind, non-inferiority SAILING study. Lancet 2013; 6736:1–9. 10.1016/S0140-6736(13)61221-0 23830355

[9] Sherman EM, Worley MV, Unger NR, Gauthier TP, Schafer JJ. Cobicistat: review of a pharmacokinetic enhancer for HIV infection. Clin Ther 2015; 37:1876–93. 26319088 10.1016/j.clinthera.2015.07.022

[10] Elliot ER, Cerrone M, Else L, et al Pharmacokinetics of dolutegravir with and without darunavir/cobicistat in healthy volunteers. J Antimicrob Chemother 2019; 74:149–56. 30272231 10.1093/jac/dky384

[11] De Nicolò A, Ianniello A, Ferrara M, et al Validation of a UHPLC-MS/MS method to quantify twelve antiretroviral drugs within peripheral blood mononuclear cells from people living with HIV. Pharmaceuticals 2021; 14:1–16. 10.3390/ph14010012 PMC7824452 33375547

[12] Cottrell ML, Hadzic T, Kashuba ADM. Clinical pharmacokinetic, pharmacodynamic and drug-interaction profile of the integrase inhibitor dolutegravir. Clin Pharmacokinet 2013; 52:981–94. 23824675 10.1007/s40262-013-0093-2 PMC3805712

[13] De Meyer S, Azijn H, Surleraux D, et al TMC114, a novel human immunodeficiency virus type 1 protease inhibitor active against protease inhibitor-resistant viruses, including a broad range of clinical isolates. Antimicrob Agents Chemother 2005; 49:2314–21. 15917527 10.1128/AAC.49.6.2314-2321.2005 PMC1140553

[14] Baril JG, Angel JB, John Gill M, et al Dual therapy treatment strategies for the management of patients infected with HIV: a systematic review of current evidence in ARV-naive or ARV-experienced, virologically suppressed patients. PLoS One 2016; 11:1–32. 10.1371/journal.pone.0148231 PMC4746196 26849060

[15] Huang Y, Huang X, Chen H, Wu H, Chen Y. Efficacy and safety of raltegravir- based dual therapy in AIDS patients: a meta-analysis of randomized controlled trials. Front Pharmacol 2019; 10:1–10. 31749699 10.3389/fphar.2019.01225 PMC6842978

[16] Cahn P, Madero JS, Arribas JR, et al Dolutegravir plus lamivudine versus dolutegravir plus tenofovir disoproxil fumarate and emtricitabine in antiretroviral-naive adults with HIV-1 infection (GEMINI-1 and GEMINI-2): week 48 results from two multicentre, double-blind, randomised, non-inferior. Lancet 2019; 393:143–55. 30420123 10.1016/S0140-6736(18)32462-0

[17] Suárez-García I, Moreno C, Ruiz-Algueró M, et al Effectiveness of the combination elvitegravir/cobicistat/tenofovir/emtricitabine (EVG/COB/TFV/FTC) plus darunavir among treatment-experienced patients in clinical practice: a multicentre cohort study. AIDS Res Ther 2020; 17:1–7. 32690099 10.1186/s12981-020-00302-2 PMC7372769

[18] Lee YL, Lin KY, Cheng SH, et al Dual therapy with dolutegravir plus boosted protease inhibitor as maintenance or salvage therapy in highly experienced people living with HIV. Int J Antimicrob Agents 2021; 58:106403. 34289404 10.1016/j.ijantimicag.2021.106403

[19] Jabłonowska E, Siwak E, Bociąga-Jasik M, et al Real-life study of dual therapy based on dolutegravir and ritonavir-boosted darunavir in HIV-1–infected treatment-experienced patients. PLoS One 2019; 14:1–10. 10.1371/journal.pone.0210476 PMC6336297 30653541

[20] Hawkins KL, Montague BT, Rowan SE, et al Boosted darunavir and dolutegravir dual therapy among a cohort of highly treatment-experienced individuals. Antivir Ther 2019; 24:513–9. 31538963 10.3851/IMP3330

[21] Capetti AF, De Socio GV, Cossu MV, et al Durability of dolutegravir plus boosted darunavir as salvage or simplification of salvage regimens in HIV-1 infected, highly treatment-experienced subjects. HIV Clin Trials 2018; 19:242–8. 30890064 10.1080/15284336.2018.1550290

[22] Vizcarra P, Fontecha M, Monsalvo M, Vivancos MJ, Rojo A, Casado JL. Efficacy and safety of dolutegravir plus boosted-darunavir dual therapy among highly treatment-experienced patients. Antivir Ther 2019; 24:467–71. 31172977 10.3851/IMP3319

[23] Agencia Española del Medicamentos y Productos Sanitarios . FICHA TECNICA PREZISTA 600 mg. Available at: http://cima.aemps.es/cima/dochtml/ft/06380002/FT_06380002.html.

[24] World Health Organization . HIV drug resistance report 2019. 2019. Available at: http://www.who.int/hiv/pub/drugresistance/hivdr-report-2019.

