# Supplementary material for: A Randomized Trial of Dolutegravir Plus Darunavir/Cobicistat as a Switch Strategy in HIV-1-Infected Patients With Resistance to at Least 2 Antiretroviral Classes
Source: Open Forum Infect Dis. 2023 Oct 31;10(11):ofad542. doi: 10.1093/ofid/ofad542 (PMC10661076; doi:10.1093/ofid/ofad542)
Supplement: ofad542_Supplementary_Data [file ofad542_supplementary_data.zip › Supplementary table 1_OFID.docx]

**Supplementary table 1.** Frequency of archived mutations in the protease and reverse transcriptase at baseline^a^

|  | **SOC arm (control), n = 44** | **2D arm (DRV/c plus DTG), n = 45** |
| --- | --- | --- |
| **Protease mutations at baseline, n (%)** |  |  |
| 10FRIVY | 20 (45.4) | 21 (46.7) |
| 11IL | - | - |
| 13V | 7 (15.9) | 7 (15.6) |
| 20MRITV | 10 (22.7) | 16 (35.6) |
| 23I | - | - |
| 24IFM | 3 (6.8) | 3 (6.7) |
| 30N | 2 (4.5) | 2 (4.4) |
| 32I | 1 (2.3) | - |
| 33IFV | 7 (15.9) | 4 (8.9) |
| 35G | 1 (2.3) | - |
| 36ILTV | 16 (36.4) | 20 (44.4) |
| 43T | 1 (2.3) | 4 (8.9) |
| 46ILV | 9 (20.5) | 10 (22.2) |
| 47AV | - | 1 (2.2) |
| 48ALMQSTV | 2 (4.5) | 7 (15.6) |
| 50LV | - | - |
| 53LY | 2 (4.5) | 2 (4.4) |
| 54AMLSTV | 7 (15.9) | 15 (33.3) |
| 58E | 2 (4.5) | 2 (4.4) |
| 60E | 4 (9.1) | 2 (4.4) |
| 62V | 9 (20.5) | 6 (13.3) |
| 63P | 32 (72.7) | 23 (51.1) |
| 71ILTV | 22 (50.0) | 19 (42.2) |
| 73ACDSTV | 7 (15.9) | 1 (2.2) |
| 74PS | 1 (2.3) | 1 (2.2) |
| 76V | - | - |
| 77I | 15 (34.1) | 9 (20.0) |
| 82ACFILMST | 13 (29.5) | 14 (31.1) |
| 83D | - | - |
| 84ACV | - | - |
| 85V | - | - |
| 88DGST | 4 (9.1) | 3 (6.7) |
| 89IMTV | 1 (2.3) | 1 (2.2) |
| 90M | 15 (34.1) | 12 (26.7) |
| 93LM | 11 (25.0) | 13 (28.9) |
| **RT mutations at baseline, n (%)** |  |  |
| 41L | 20 (45.4) | 20 (44.4) |
| 44AD | 1 (2.3) | 5 (11.1) |
| 62V | 3 (6.8) | 6 (13.3) |
| 65NER | 5 (11.4) | 9 (20.0) |
| 67EGHNSTdel | 26 (59.1) | 18 (40.0) |
| 68del | - | - |
| 69DGNinser | 7 (15.9) | 7 (15.6) |
| 70EGQNRSTdel | 12 (27.3) | 12 (26.7) |
| 74IV | 5 (11.4) | 2 (4.4) |
| 75AILMST | 9 (20.5) | 6 (13.3) |
| 77L | 1 (2.3) | 1 (2.3) |
| 90I | 2 (4.5) | 2 (4.4) |
| 98G | 5 (11.4) | 8 (17.8) |
| 100IV | 6 (13.6) | 4 (8.9) |
| 101EHNPQ | 7 (15.9) | 11 (24.4) |
| 103EHNQRS | 32 (72.7) | 28 (62.2) |
| 106AIMT | 1 (2.3) | 5 (11.1) |
| 108I | 2 (4.5) | 3 (6.7) |
| 115F | 3 (6.8) | - |
| 116Y | - | 1 (2.2) |
| 118I | 9 (20.5) | 8 (17.8) |
| 138AGKQR | 2 (4.5) | 3 (6.7) |
| 151LM | - | 2 (4.4) |
| 179DEFTL | 7 (15.9) | 5 (11.1) |
| 181CFGISV | 7 (15.9) | 13 (28.9) |
| 184IV | 32 (72.7) | 29 (64.4) |
| 188CFHL | 1 (2.3) | 5 (11.1) |
| 190ACEQSTV | 7 (15.9) | 12 (26.7) |
| 210FSW | 15 (34.1) | 17 (37.8) |
| 215ACDEFILNSVY | 28 (63.6) | 27 (60.0) |
| 219ENQRW | 16 (36.4) | 13 (28.9) |
| 221Y | 1 (2.3) | 3 (6.7) |
| 225H | 3 (6.8) | 3 (6.7) |
| 227CILVR | 1 (2.3) | 2 (4.4) |
| 230IL | - | - |
| 234I | - | - |
| 236I | - | - |
| 238NT | - | - |
| 318F | - | - |
| 348I | - | - |

^a^Stanford DB, version 9.0, available at: https://hivdb.stanford.edu

Abbreviations: PR, protease; DTG, dolutegravir; DRV/c, darunavir/cobicistat; RT, reverse transcriptase
