# Supplementary material for: A Randomized Trial of Dolutegravir Plus Darunavir/Cobicistat as a Switch Strategy in HIV-1-Infected Patients With Resistance to at Least 2 Antiretroviral Classes
Source: Open Forum Infect Dis. 2023 Oct 31;10(11):ofad542. doi: 10.1093/ofid/ofad542 (PMC10661076; doi:10.1093/ofid/ofad542)
Supplement: ofad542_Supplementary_Data [file ofad542_supplementary_data.zip › Supplementray table 2_OFID.docx]

**Supplementary table 2**. Number of antiretroviral drugs prior to randomization

|  | **SOC arm (control), n = 44** | **2D arm (DRV/c plus DTG), n = 45** | **Total, n=89** |
| --- | --- | --- | --- |
| **Protease inhibidors, n (%)** |  |  |  |
| Darunavir^a^ | 34 (77.3) | 41 (91.1) | 75 (84.2) |
| Lopinavir/ritonavir | 26 (59.1) | 26 (57.8) | 52 (58.4) |
| Atazanavir^c^ | 19 (43.2) | 12 (26.7) | 31 (34.8) |
| Tipranavir/ritonavir | 4 (9.1) | 5 (11.1) | 9 (10.1) |
| Nelfinavir | 15 (34.1) | 22 (48.9) | 38 (42.7) |
| Saquinavir^b^ | 16 (36.4) | 12 (26.7) | 28 (31.4) |
| Amprenavir/  FosAmprenavir/ritonavir | 7 (15.9) | 8 (17.8) | 15 (16.9) |
| Indinavir^b^ | 21 (47.7) | 15 (33.3) | 36 (40.4) |
| Ritonavir | 15 (34.1) | 19 (42.2) | 34 (38.2) |
|  |  |  |  |
| **NRTI, n (%)** |  |  |  |
| Zidovudine | 34 (77.3) | 38 (84.4) | 75 (84.2) |
| Estavudine | 37 (84.1) | 35 (77.8) | 72 (80.9) |
| Lamivudine/Emtricitabine | 42 (95.5) | 45 (100.0) | 87 (97.8) |
| Abacavir | 35 (79.5) | 40 (88.9) | 75 (84.2) |
| Tenofovir^d^ | 42 (95.5) | 41 (91.1) | 83 (93.3) |
| Zalcitabine | 14 (31.8) | 15 (33.3) | 29 (32.6) |
| Didanosine | 29 (65.9) | 34 (75.6) | 63 (70.8) |
| **NNRTI, n (%)** |  |  |  |
| Efavirenz | 23 (52.3) | 21 (46.7) | 44 (49.4) |
| Nevirapine | 23 (52.3) | 25 (55.6) | 48 (53.9) |
| Etravurune | 21 (47.7) | 21 (46.7) | 42 (47.2) |
| Rilpivirine | 7 (15.9) | 4 (0.9) | 11 (12.4) |
|  |  |  |  |
| **Fusion inhibidors/**  **CCR5 antagonists, n (%)** |  |  |  |
| Enfuvirtide | 5 (11.4) | 7 (15.6) | 12 (13.5) |
| Maraviroc | 10 (22.7) | 6 (13.3) | 16 (17.9) |
|  |  |  |  |
| **Integrase inhibidors, n (%)** |  |  |  |
| Raltegravir | 26 (59.1) | 30 (66.7) | 56 (62.9) |
| Elvitegravir/cobicistat | 8 (18.2) | 3 (6.7) | 11 (12.4) |
| Dolutegravir | 15 (34.1) | 17 (37.8) | 32 (35.9) |
| Bictegravir | - | 1 (2.2) | 1 (1.1) |

^a^Including co-formulations with ritonavir and cobicistat as boosters

^c^Including unboosted atazanavir and co-formulations with ritonavir and cobicistat

^b^Including unboosted formulation and ritonavir as booster

^d^Including tenofovir disoproxil fumarate and tenofovir alafenamide;

Abbreviations: DTG, dolutegravir; DRV/c, darunavir/cobicistat; RT, reverse transcriptase; NNRTI, non-nucleoside reverse transcriptase inhibitor
