# Supplementary material for: A Randomized Trial of Dolutegravir Plus Darunavir/Cobicistat as a Switch Strategy in HIV-1-Infected Patients With Resistance to at Least 2 Antiretroviral Classes
Source: Open Forum Infect Dis. 2023 Oct 31;10(11):ofad542. doi: 10.1093/ofid/ofad542 (PMC10661076; doi:10.1093/ofid/ofad542)
Supplement: ofad542_Supplementary_Data [file ofad542_supplementary_data.zip › Supplementary Table 3_OFID_cleaned (v2).docx]

**Supplementary table 3**. Clinical data from subjects experiencing virological failure.

| **Study ID** | **Allocation** | **Historical genotyping tests at baseline** | **Time on virological suppression before randomization (weeks)** | **Time on the last treatment before randomization (weeks)** | **Optimised ART at baseline** | **HIV-1 RNA at VF (copies/mL)** | **HIV-1 RNA at VF confirmation (copies/mL)** | **Time to VF (weeks)** | **ART adherence at VF** | **Trough drug concentrations in plasma** | **Follow-up after VF** | **Genotype at VF**^a^ |
| --- | --- | --- | --- | --- | --- | --- | --- | --- | --- | --- | --- | --- |
| 8-6 | SOC arm | RT: M41L, A62V, D67N, K70R, V75I, L100I, K103N, M184V, T215Y, K219Q, P225H  PR: no mutations | 107 | 145 | DRV/c + RAL 400 mg twice daily + ETR twice daily | 83 | 89 | 12 | 100% | DRV: 232.1 ng/mL  COBI: <LLD | Loss of follow-up.  Changes in ART were not performed. | PR: no mutations detected  IN: no mutations detected  RT: not amplified |
| 1-27 | SOC arm | RT: M41L, K103NQ, M184V, L210W and T215SY  PR: L10I, I13V, L63P, V77I | 172 | 44 | DRV/c + FTC/TAF | 74 | 2,115 | 24 | Week 12: 43%  Week 24: Voluntary ART interruption | DRV: 4,819.3 ng/mL  COBI: 627.4 ng/mL | ART counselling.  Changes in ART were not performed.  Resuppression of HIV-1 was achieved at week 36 (VL <50 copies/mL). | Not amplified |

^a^Genotyping test was performed at VF confirmation (second determination of HIV-1 RNA).

Abbreviations: SOC, standard of care; RT, reverse transcriptase; PR, protease; ART, antiretroviral treatment, DRV/c, darunavir/cobicistat; RAL, raltegravir; ETR, etravirine; FTC, emtricitabine; TAF, tenofovir alafenamide; VF, virological failure; COBI, cobicistat; VL, viral load; LLD, low limit of detection.
