# Supplementary figures and images for: A Randomized Trial of Dolutegravir Plus Darunavir/Cobicistat as a Switch Strategy in HIV-1-Infected Patients With Resistance to at Least 2 Antiretroviral Classes
Source: Open Forum Infect Dis. 2023 Oct 31;10(11):ofad542. doi: 10.1093/ofid/ofad542 (PMC10661076; doi:10.1093/ofid/ofad542)

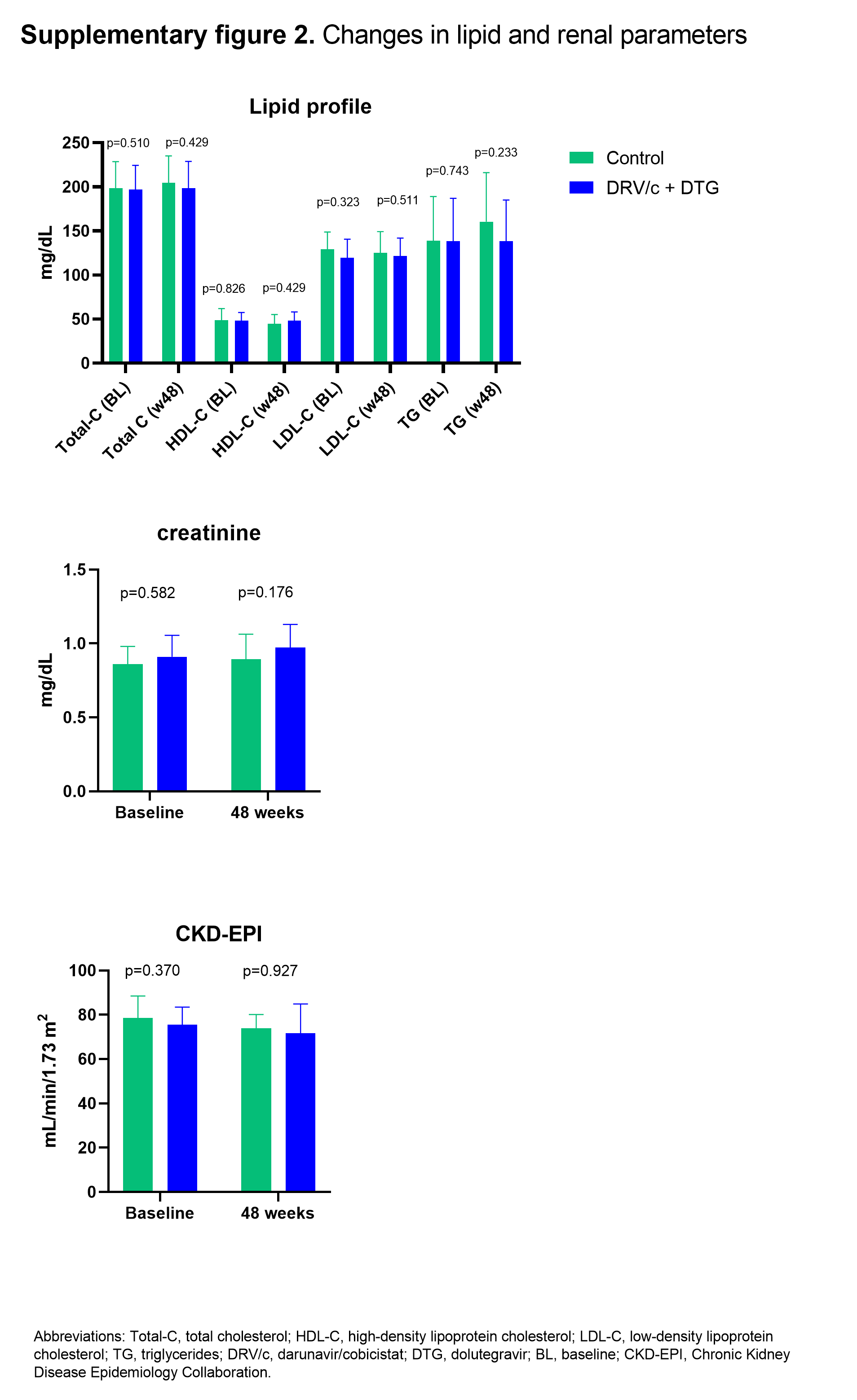

Supplement: ofad542_Supplementary_Data [file ofad542_supplementary_data.zip › Supplementary fig 2 2D_OFID.tif]

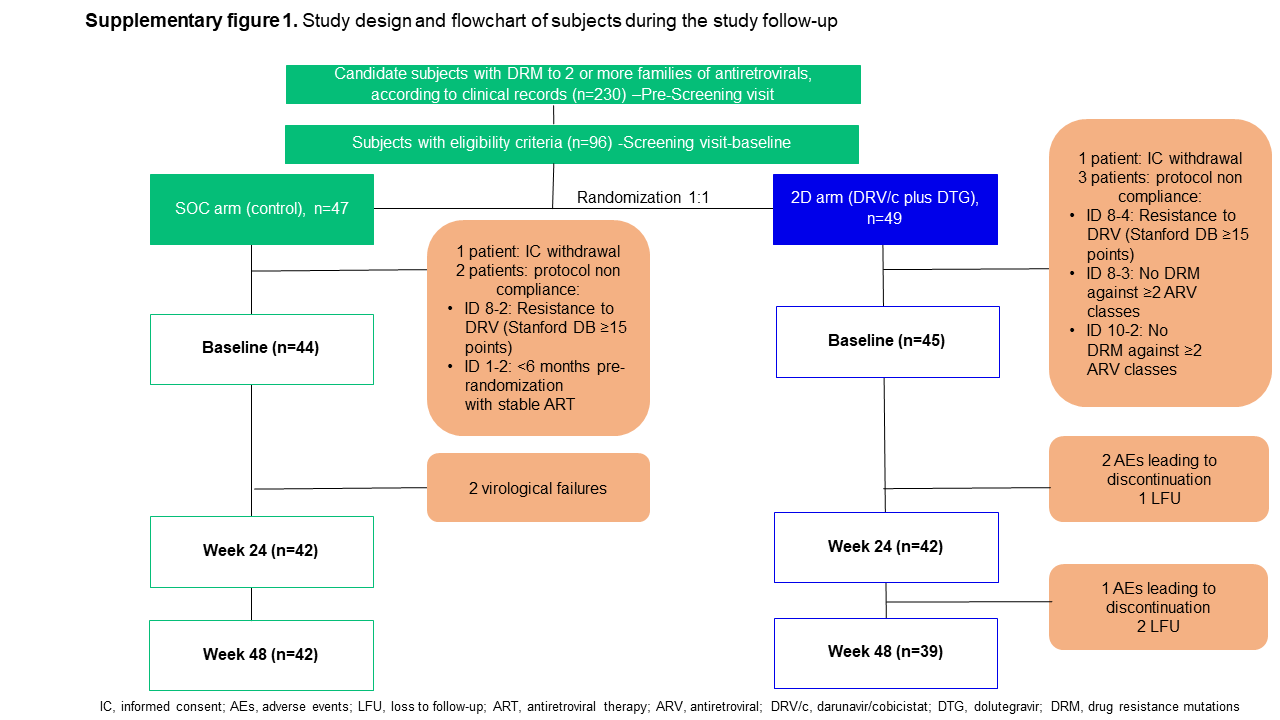

Supplement: ofad542_Supplementary_Data [file ofad542_supplementary_data.zip › Supplementary Figure 1_2D_OFID 170923.tif]
